# CD4+CD45RA+ T-cells from early diffuse systemic sclerosis patients produce high levels of interleukin-17

**DOI:** 10.1186/1479-5876-9-S2-P62

**Published:** 2011-11-23

**Authors:** Lenny van Bon, Marta Cossu, Wim van den Berg, Madelon Vonk, Hans Koenen, Timothy Radstake

**Affiliations:** 1 Dept. of Rheumatology, Radboud University Nijmegen Medical Centre, The Netherlands; 2 Dept. of Blood Transfusion and Transplantation Immunology, Radboud University Nijmegen Medical Centre, The Netherlands

## Background

In Systemic Sclerosis (SSc) the detrimental role of the immune system is a topic of great interest. In recent genetic association studies most genes identified to date are immunity related genes. A contribution of T-cells as important cytokine producing cells is shown and the skewing of this population can be an important player in the maintenance and progression of inflammation and fibrosis.

## Material and methods

After inclusion 20 SSc patients were stratified as having diffuse or limited disease according to the extent of skin involvement. Patients were further stratified as having early or late disease, early being less then 2 years after the start of the first non-Raynaud symptom. After isolation from 60 ml of venous blood CD4+ T-cells were divided in CD45RO and CD45RA by MACS bead isolation. Cells were stimulated with PMA and iomycin. Cytokine levels were measured in supernatant using the Luminex Bead Array.

## Results

The IL-17 production was clearly increased in the supernatant of CD4+CD45RO cells but even more clearly in the CD4+CD45RA cells from early diffuse SSc patients compared to healthy controls or limited SSc patients. Limited SSc patients showed a clear decrease in IL-10 and interferon-γ production.

## Conclusions

The CD4+CD45RA+ T-cells from early diffuse SSc patients produce increased levels of IL-17 suggestion a more pro-inflammatory phenotype in this T-cell subset. Together with a normal production of IL-13 this could create a proinflmmatory and profibrotic condition in this subset of patients. The decreased production of IL-10 and interferon-γ in limited SSc patients shows a decrease of Th1 and Treg involvement in this disease subset. A prospective study must show if these findings could contribute to the assessing the prognosis and the treatment of SSc patients.

